# Molecular Electron Density Theory: A Modern View of Reactivity in Organic Chemistry

**DOI:** 10.3390/molecules21101319

**Published:** 2016-09-30

**Authors:** Luis R. Domingo

**Affiliations:** Department of Organic Chemistry, University of Valencia, Dr. Moliner 50, Burjassot, Valencia E-46100, Spain; domingo@utopia.uv.es; www.luisrdomingo.com

**Keywords:** molecular electron density theory, DFT reactivity indices, electron localisation function, non-covalent interactions, electron density, molecular mechanisms, chemical reactivity

## Abstract

A new theory for the study of the reactivity in Organic Chemistry, named Molecular Electron Density Theory (MEDT), is proposed herein. MEDT is based on the idea that while the electron density distribution at the ground state is responsible for physical and chemical molecular properties, as proposed by the Density Functional Theory (DFT), the capability for changes in electron density is responsible for molecular reactivity. Within MEDT, the reactivity in Organic Chemistry is studied through a rigorous quantum chemical analysis of the changes of the electron density as well as the energies associated with these changes along the reaction path in order to understand experimental outcomes. Studies performed using MEDT allow establishing a modern rationalisation and to gain insight into molecular mechanisms and reactivity in Organic Chemistry.

## 1. Structure and Reactivity in Organic Chemistry Based on Quantum Chemical Models

*Why* and *how* do reactions take place are two fundamental questions in Organic Chemistry. Many chemical concepts have been developed through a large number of experiments, however, understanding how molecules change is by no means experimentally easy because changes take place in a very short time. Based on experiments, some important theories, such as the transition state theory [1], have been developed in kinetic chemistry, which have permitted to establish fundamental concepts used in the study of molecular mechanisms. Within this theory, the concept of the activation complex or transition state structure (TS) enabled the establishment of a relationship between the experimental activation energy [2] and the energy of the TS associated with an organic reaction.

Since the introduction of the chemical bond concept by Lewis in 1916 [3], two different quantum mechanics theories to explain chemical bonding, namely, the valence bond theory [4,5,6] (VBT) and the molecular orbital theory [7] (MOT), were established. The two theories were developed at about the same time, but quickly diverged into two different schools that have established a complete interpretation of chemistry [8]. Both theories are based on the resolution of Schrödinger’s equation [9] (see Equation (1)). The information obtained from the resolution of Schrödinger’s equation is a wavefunction Ψ whose square describes the electron density distribution around the nuclei, and the total electronic energy E associated with this wavefunction Ψ. The square of the wavefunction Ψ is related with the electron density, which is a physically observable [10].

*H*·Ψ = E·Ψ (Schrödinger equation) (1)

Until the mid-1950s, chemistry was dominated by the classical VBT, which expresses the molecular wavefunction Ψ as a combination of explicit covalent and ionic structures based on pure atomic orbitals (AOs) or hybrid atomic orbitals. However, the computational effort required to perform *ab initio* calculations in classical VBT made it to be employed in an oversimplified manner. Relevant concepts used in Organic Chemistry such as hybridisation and resonance were developed within VBT. At the same time when this early *ab initio* VBT lacked accuracy and did not progress, MOT included efficient implementations, which have provided the chemical community with computational software of ever-increasing speed and capabilities [8].

Due to its complexity, the mathematical resolution of Schrödinger’s equation is only feasible for very small systems such as the helium atom, the dihydrogen cation or the hydrogen molecule, but not possible for organic molecules containing few carbon atoms. Therefore, a series of physical/mathematical approaches was developed for the study of molecules using quantum chemical procedures. One of them was developed within MOT, in which the polyelectronic wavefunction Ψ of Equation (1) is approximated as an antisymmetrised product of a series of monoelectronic orbitals Ψ_i_ named molecular orbitals (MOs). In addition, these MOs were obtained by a linear combination of atomic orbitals (LCAO), which are mathematical functions describing the electron movement in a hydrogen-type AO φ_j_ [11]. In order to improve MOs, a series of mathematical functions, known as basis functions, were further developed. However, the standard polarised or diffuse functions [12], which generate more complete basis sets, have a quite different physical meaning than when the minimal basis set is used. In MOT, electrons are not assigned to individual bonds between atoms, but they are treated as moving under the influence of the nuclei along MOs in the whole molecule.

The simplest approach to MOT used in Organic Chemistry is Hückel’s molecular orbital theory [13,14,15,16] (HMOT) established in 1930, in which only valence AOs are used to build MOs. This very simple approach, which is widely used in Organic Chemistry nowadays, was employed in the 1950s to establish important organic reactivity models such as the Frontier Molecular Orbital (FMO) theory [17] for the study of the reactivity in Organic Chemistry [18].

Molecular geometry optimisations based on quantum chemical procedures allow obtaining accurate geometries that can be contrasted with those obtained by experimental X-ray data. These quantum chemical procedures do not only permit to obtain accurate structures for reactants and products, but also for TSs and intermediates involved in organic reactions.

In the two last decades of the 20th century, *ab initio* Hartee-Fock (HF) calculations [12] enabled the study of the molecular mechanism of many organic reactions by characterising the stationary points involved in a reaction: i.e., reagents, TSs, intermediates and products. While HF calculations rendered good geometries, experimental activation energies were overestimated. Consequently, very time-consuming post-HF energy calculations [12] were demanded to reproduce the experimental values.

At the end of the 20th century, based on the theorems by Hohenberg and Kohn, a new theory to study the electronic structure of the matter, known as Density Functional Theory (DFT) [19], in which the ground state (GS) energy of a nondegenerated N electron system is only a functional of the density *ρ*(**r**), was developed (see Equation (2)). Similar to the quantum chemical theories based on Schrödinger’s equation, the resolution of the functional of the electron density *ρ*(**r**) for a complex system is neither computationally feasible. As an approximation, the Kohn–Sham equations [20] were introduced in analogy to the HF equation. However, in the DFT framework, the Kohn–Sham orbitals do not define any wavefunction although they are used to calculate the one-electron density distribution function. In any case, it is important to highlight that MOs are not physical observables but only mathematical constructs that cannot be determined by experiment [21]. In contrast, the electron density distribution in a molecule or crystal can be observed by electron diffraction and X-ray crystallography [22,23]; and it can also, and often more readily, be obtained from *ab initio* or DFT calculations. In the last decades, a series of empiric DFT functionals such as B3LYP [24,25], MPWB1K [26] and, more recently, M06-2X [27], which provide accurate energies, have been developed, making the study of organic reactions with a computational demand similar to HF calculations possible.

E[*ρ*(**r**)] = *∫ρ*(**r**)*v*(**r**) *d***r** + F[*ρ*(**r**)]  (Hohenberg-Kohn equation) (2)

Organic reactions involving the formation of C–C single bonds are the most significant ones within the wide arsenal of reactions used in organic synthesis since they enable the building of more complex molecules. Among the diverse possibilities of creating a new C–C single bond, those involving the participation of C=C double bonds are the most attractive methods.

**Scheme 1:**
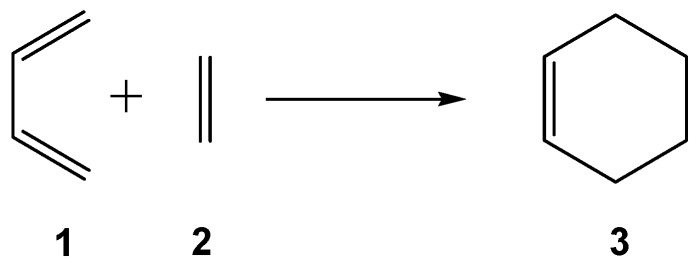
DA reaction between butadiene **1** and ethylene **2** giving cyclohexene **3**.

The Diels–Alder (DA) reaction [28] between butadiene **1** and ethylene **2** (see Scheme 1), which has theoretically been widely studied, can be used as a test of different quantum chemical approaches (see Table 1).

As can be observed in Table 1, both reaction energies, ΔE_reac_ in the range from 36.0 (HF) to 57.7 (MPWB1K) kcal·mol^−1^, and activation energies, ΔE_act_ in the range from 19.4 (M06-2X) to 47.4 (HF) kcal·mol^−1^, vary in a wide range. Note that the predicted B3LYP activation energy, 24.8 kcal·mol^−1^, was found to be closer to that experimentally estimated, 27.5 kcal·mol^−1^, while the M06-2X activation energy, 19.4 kcal·mol^−1^, is underestimated. Note that inclusion of thermal corrections to the electronic energies increases the activation enthalpies by ca. 2 kcal·mol^−1^.

**Figure 1:**
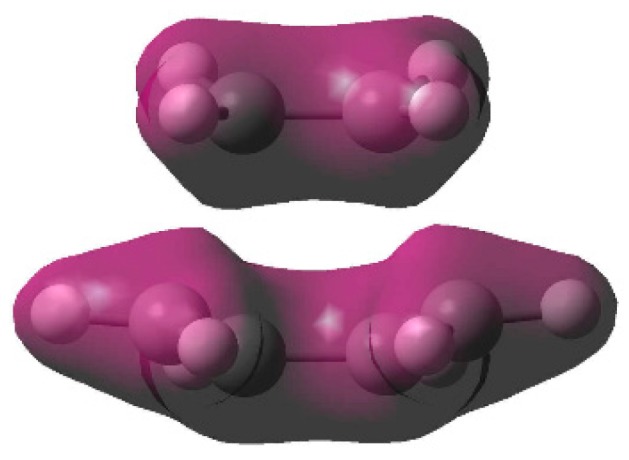
B3LYP/6-31G(d) map of the total electron density of the transition state structure **TS1** associated with the DA reaction between butadiene **1** and ethylene **2**. Isovalues of the map of the electron density are 0.08.

Interestingly, in spite of these wide ranges of activation energies, TS geometries are found to be non-dependent on the quantum chemical model. Thus, in all these cases, the distance between the two carbons involved in the formation of the new C–C single bonds in the synchronous **TS1** is found to be ca. 2.2 Å (see Table 1). That is, while the computed activation energies for this reaction are found to be very dependent on the computational method, the TSs geometries, which depend on their electronic structures, proved to be very similar. Note that the relative position of the atoms in a molecule depends on the electronic interactions between nuclei and electrons; i.e., geometries depend on the total molecular electron density. Consequently, all computational approaches presented in Table 1 afford a very similar electron density distribution, indicating a very similar bonding pattern of **TS1** (see Figure 1).

Unfortunately, the direct analysis of the total electron density obtained through these quantum chemical procedures does not provide any chemical information such as the bonding pattern of the molecular structure. This information is more significant for TS geometries as it allows understanding the evolution of the bonding changes in these critical structures.

## 2. Quantum Chemical Topological Analysis of the Electron Density and Molecular Mechanisms

In spite of the advances made in quantum chemical theory, the characterisation of chemical bonds, and more specifically the breaking/forming processes along a reaction, appeared to be unresolved [29]. Like many other chemical concepts, chemical bonds are defined in a rather ambiguous manner as they are not observable, but rather belong to a representation of the matter at a microscopic level which is not fully consistent with quantum mechanical principles. To harmonise the chemical description of matter with quantum chemical postulates, several mathematical models have been developed. Among them, the theory of dynamical systems [30], convincingly introduced by Bader in the early 1960s through the theory of atoms in molecules (AIM) [31,32], has become a powerful method of analysis. The AIM theory enables a partition of the electron density within the molecular space into basins associated with atoms. The development of the AIM theory was the origin of a significant contribution to conceptual chemistry in the definition of concepts such as the atom inside a molecule or bond critical points [33,34].

Another appealing procedure, introduced in 1990, that provides a more straightforward connection between the electron density distribution and the chemical structure is the quantum chemical analysis of the electron localisation function (ELF) of Becke and Edgecombe [35]. ELF constitutes a useful relative assessment of the electron pair localisation characterising the corresponding electron density. Within the framework of DFT, ELF is a density-based property that can be interpreted in terms of the positive-definite local Pauli and Thomas Fermi kinetic energy densities in the given system. In the validity of such a framework, these quantities provide key information to evaluate the relative local excess of kinetic energy density associated with the Pauli principle. ELF values are situated in the [0, 1] range, the highest values being associated with the spatial positions with higher relative electron localisation [35].

In this sense, Silvi and Savin presented in 1991 the ELF in a very chemical fashion, using their topological analysis as an appealing model of chemical bonding [36,37,38,39]. After an analysis of the electron density, ELF divides the electron density of a molecule into basins, i.e., domains in which the probability of finding an electron pair is maximal. Basins are classified as core basins and valence basins. The latter are characterised by the synaptic order, i.e., the number of atomic valence shells in which they participate [40]. Thus, there are monosynaptic, disynaptic, trisynaptic basins and so on. Monosynaptic basins, labelled V(A), correspond to lone pairs or non-bonding regions, while disynaptic basins, labelled V(A,B), connect the core of two nuclei, namely A and B, and, thus, correspond to a bonding region between A and B. This description recovers the Lewis bonding model, providing a very suggestive graphical representation of the molecular system.

The characterisation of the electron density reorganisation to evidence the bonding changes along a reaction path is the most attractive method to characterise a reaction mechanism [41,42,43]. To perform these analyses quantitatively, the bonding evolution theory (BET), consisting of the joint-use of ELF topology and Thom’s catastrophe theory [44,45,46] (CT) was proposed by Krokidis et al. [47] in 1997 as a new tool for analysing the electronic changes in chemical processes, being widely applied in the study of different elementary reactions [48,49,50,51,52,53,54,55,56,57].

**Figure 2:**
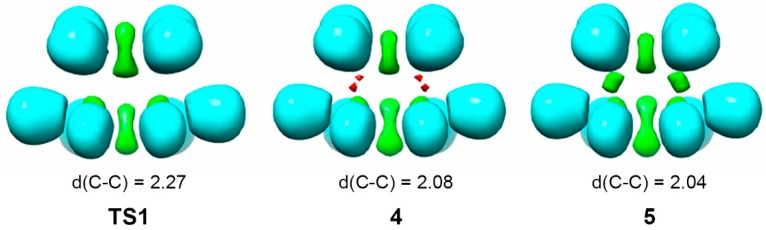
ELF valence basins in **TS1** and in the structures defining *phase V*, structure **4**, and *phase VI*, structure **5**, involved in the synchronous C–C single bond formation in the non-polar DA reaction between butadiene **1** and ethylene **2**. The pseudoradical centres involved in the C–C bond formation are characterised by the four V(C) monosynaptic basins depicted in red in structure **4**. ELF valence basins were represented at an isosurface value of 0.8 and C–C distances are given in Angstroms. Calculations were performed at the B3LYP/6-31G(d) level.

The BET study of the DA reaction between butadiene **1** and ethylene **2** makes it possible to obtain three appealing conclusions [41]: (i) the seven phases characterizing the one-step mechanism permitt to establish that the bonding changes involved in this DA reaction are non-concerted [58], thus ruling out the pericyclic mechanism [59,60]; (ii) ELF topological analysis of the electron density of **TS1** shows that there is no bonding region between the C1 and C6, and C4 and C5 atoms (see **TS1** in Figure 2) in clear agreement with the electron density distribution of **TS1** given in Figure 1; and (iii) the formation of the C–C single bonds takes place after passing **TS1** at a C–C distance of 2.04 Å (see **5** in Figure 2) [61].

## 3. Global Electron Density Transfer (GEDT) and Activation Energy of Polar Organic Reactions

In 1995, when I started the theoretical studies of reaction mechanisms, an important question was posed: what is the origin of the activation energies associated with the TSs of organic reactions? Unfortunately, the answer to this question cannot be obtained from the analysis of the energy and geometrical parameters. The response was obtained after analysing a large number of theoretical studies devoted to reaction mechanisms.

A DFT study of the hetero-DA reactions of nitroethylene **6**, CH_2_=CH–NO_2_, with three ethylenes of increased nucleophilic character, namely, propene **7a**, CH_2_=CH–CH_3_, methyl vinyl ether **7b**, CH_2_=CH–OCH_3_, and *N*,*N*-dimethylvinyl amine **7c**, CH_2_=CH–N(CH_3_)_2_, showed a good correlation between the activation energies and the nucleophilic/electrophilic behaviours of the reagents, i.e., in this short series, the increase of the nucleophilic character of the ethylene CH_2_=CH–G decreased the activation energy associated with the C–C bond formation [62].

Interestingly, a good correlation between the polar character of the reactions, measured by the global electron density transfer [61] (GEDT) at the TSs, and their feasibility was established. Along a polar reaction, there is a GEDT from the nucleophile to the electrophile; the larger the GEDT at the TS is, the more polar the reaction. The GEDT at the TSs is computed by sharing the natural atomic charges obtained by a natural population analysis (NPA) [63,64] between the nucleophilic and the electrophilic frameworks [65]. The GEDT concept comes from the observation that the electron density transfer that takes place from the nucleophile to the electrophile along polar reactions is not a local process, but a global one involving the two molecules [66]. Consequently, the whole system becomes one entity from the beginning of the reaction being, therefore, structurally indivisible. GEDT values are hardly dependent on the computational method used to obtain the atomic charges as these values come from the integration of the electron density of two molecular frameworks that are usually not yet bound at the TSs [67].

The good correlation between the polar character of the reactions and their feasibility was quantitatively ascertained in 2002 analysing the Parr electrophilicity ω index [68] of reagents involved in DA reactions [69]. This first DFT study involving simple organic molecules participating in DA reactions enabled the establishment of a single electrophilicity scale, in which both dienes and dienophiles were included [69], thus permitting the establishment of an adequate correlation between the difference of the electrophilicity ω indices of the reagents, Δω, and the feasibility of the DA reactions [69]. The more electrophilic a reagent is, i.e., when it is located at the top of the scale, and more nucleophilic the other reagent is, i.e., when it is located at the bottom of the scale, the more polar and faster the reaction. However, in complex organic molecules having more than one functional group, the nucleophilicity cannot be correlated with the inverse of the electrophilicity ω index [70,71]. Consequently, to analyse the polar character of an organic reaction, the electrophilicity ω index and the nucleophilicity *N* index [70,71], introduced in 2008, of the two reagents should be analysed.

**Scheme 2:**
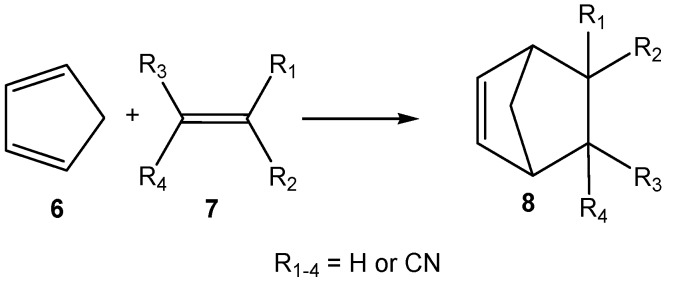
DA reactions between Cp **6** and the cyanoethylene series **7** giving the series of cyclohexene derivatives **8**.

**Figure 3:**
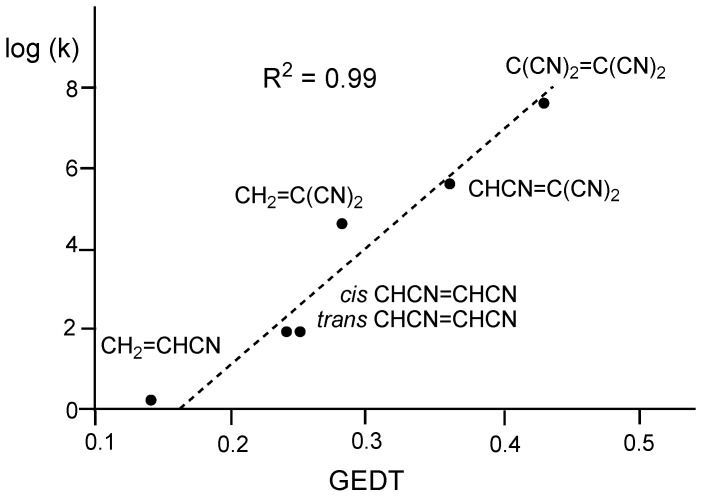
Plot of the logarithm of the experimental rate constant k vs GEDT, in e, for the DA reactions of Cp **6** with the cyanoethylene series **7**; R^2^ = 0.99 [72]. Calculations were performed at the B3LYP/6-31G(d) level.

A further DFT study of the DA reactions between cyclopentadiene (Cp) **6** and the cyanoethylene series **7** (see Scheme 2) [72], experimentally studied by Sauer [73,74], allowed establishing a very good correlation between the GEDT found at the TSs and the logarithm of the experimental rate constants, indicating that the GEDT could be one of the key factors in the activation energy [65] (see Figure 3). This finding allowed us to establish a pertinent classification of DA reactions in non-polar, polar and ionic DA reactions [65].

What is the origin of the GEDT in organic reactions? The electronic chemical potential μ [75], defined within the conceptual DFT [76,77] as μ = (∂E/∂N)_v_, is associated with the feasibility of a molecule to exchange electron density with the environment at the GS. Sanderson proposed the electronegativity equalisation principle [78], in chemistry, according to which electronegativity χ [79], which is the negative of electronic chemical potential μ, tends to equalise. Consequently, when two molecules A and B, with μ_A_ > μ_B_, approach each other, there is a flux of electron density from A, the less electronegative species, towards B, the more electronegative one, to equilibrate the electronic chemical potential μ_AB_. The larger the electronic chemical potential difference, Δμ_A−B_, the larger the GEDT.

## 4. GEDT Favours the Electron Density Changes Required to Reach the TS Structure

After finding this interesting relationship between polarity of the reaction and activation energy, an appealing issue remained unresolved: how can GEDT modify the pattern in bonding changes along polar reactions? That is, how can the polarity of the reaction modify the activation energy? To answer these questions, an ELF topological analysis of the bonding changes, i.e., electron density changes, along a reaction path, as well as an analysis of the energies associated with these bonding changes is required.

ELF topological analysis along a large number of organic reactions involving the participation of C=C double bonds made it possible to establish two appealing conclusions about the C–C bond formation in these organic reactions [61]: (i) non-polar, polar and ionic reactions present a similar pattern for the formation of C–C single bonds, characterised by a C-to-C coupling of two pseudoradical centres [80,81] at a C–C distance in the narrow range from 2.0 to 1.9 Å. These pseudoradical centres are generated along the reaction path; (ii) while along non-polar reactions the pseudoradical centres are generated by the homolytic rupture of the C=C double bonds present in the two reagents, in polar and ionic reactions the formation of these pseudoradical centres are favoured by the GEDT that takes place from the nucleophile to the electrophile. Consequently, *the GEDT taking place along the reaction path in polar and ionic reactions causes significant changes in the electron density distribution in both nucleophilic and electrophilic species*.

In non-symmetric molecules, a non-symmetric electron density distribution takes place during the GEDT process. Thus, while in the nucleophilic species some atoms lose less electron density, in the electrophilic species some atoms gather more electron density. These relevant atoms correspond to the most nucleophilic and most electrophilic centres of the molecules, which are properly characterised by the use of the proposed nucleophilic $P_{k}^{-}$ and electrophilic $P_{k}^{+}$ Parr functions [82,83]. The non-symmetric electron density distribution is responsible for the chemo- and regioselectivity found in polar reactions involving non-symmetric molecules, since in these reactions the most favourable reaction channels correspond to those with the most favourable nucleophilic/electrophilic two-centre interaction, i.e., between the atoms in which the two pseudoradical centres generated through the GEDT will be formed.

**Figure 4:**
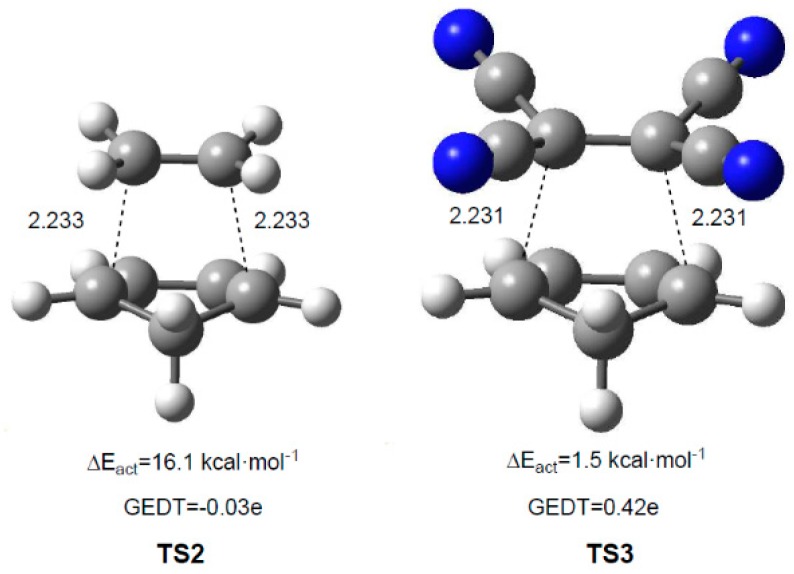
MPWB1K/6-31G(d) geometries and lengths of the forming bonds, in Angstroms, activation energies, in kcal·mol^−1^, and GEDT values, in e, of the TSs involved in the non-polar DA reaction between Cp **6** and ethylene **2**, **TS2**, and in the polar DA reaction between Cp **6** and TCE **9**, **TS3**.

In order to prove how the GEDT reduces the activation energy in polar reactions, the TSs associated with the non-polar DA reaction between Cp **6** and ethylene **2**, **TS2**, and the polar DA reaction between Cp **6** and TCE **9** (**7**, R_1_ = R_2_ = R_3_ = R_4_ = CN), **TS3**, which is the most polar reaction of the cyanoethylene series (see Scheme 2), are analysed. The corresponding activation energies, TS geometries and GEDT at the TSs are given in Figure 4.

Similar to the non-polar DA reaction between butadiene **1** and ethylene **2**, the reaction between Cp **6** and ethylene **2** presents a high activation energy, 16.1 kcal·mol^−1^. Note that ethylene **2** has been classified as a marginal electrophile and as a marginal nucleophile, not being able to participate in polar processes [69]. On the other hand, TCE **9** is one of the most electrophilic ethylenes [69]. This fact, together with the high nucleophilic character of Cp **6**, causes this polar DA reaction not to have an appreciable barrier, 1.5 kcal·mol^−1^. Consequently, a decrease of ca. 15 kcal·mol^−1^ is observed in this polar DA reaction. Non-polar **TS2** does not present any appreciable GEDT value, −0.03 e, while polar **TS3** presents a high GEDT value, 0.42 e. A comparison of the geometrical parameters of these TSs indicates that the lengths of the C–C forming single bonds at both TSs are very similar, 2.233 Å (**TS2**) and 2.231 Å (**TS3**). Consequently, it is expected that the distribution of the total electron density in both TSs will be similar, and therefore, they will have a similar bonding pattern.

**Figure 5:**
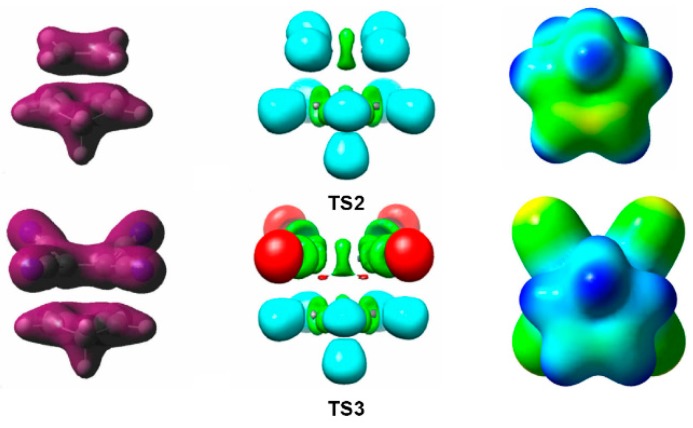
MPWB1K/6-31G(d) maps of the total electron density and ELF valence basins, and MEP for the TSs involved in the non-polar DA reaction between Cp **6** and ethylene **2**, **TS2**, and in the polar DA reaction between Cp **6** and TCE **9**, **TS3**. Isovalues of the map electron density are 0.08 and ELF valence basins were represented at an isosurface value of 0.8.

Hence, where is the electronic difference between both TSs? ELF topological analysis of the electron density of the TS associated with the non-polar DA reaction between Cp **6** and ethylene **2**, see **TS2** in Figure 5, resembles that of the TS associated with the non-polar DA reaction between butadiene **1** and ethylene **2**, see **TS1** in Figure 1. ELF topological analysis of **TS2** indicates that while the C–C double bonds of Cp **6** and ethylene **2** are already broken, formation of the four pseudoradical centres has not begun yet. Consequently, the high activation energy associated with these non-polar TSs has been related to the energy cost associated with the depopulation of the C–C double bond regions, i.e., the rupture of the C–C double bonds, present in the diene and in the dienophile [84]. Interestingly, ELF topological analysis of **TS3** shows a clear difference with that of **TS2**; two V(C) monosynaptic basins integrating 0.46 e appear at the two ethylenic carbons of TCE **9** (see the two V(C) monosynaptic basins in red in Figure 5). These monosynaptic basins, which are required for the subsequent C–C single bond formation, are formed at the most electrophilic centres of TCE **9** as a consequence of the large GEDT taking place in this polar DA reaction, GEDT = 0.43 e. Consequently, it appears that the GEDT taking place from the nucleophile to the electrophile in a polar process favours the changes in electron density required to reach the TS geometry (see the different colour of the Cp framework in the molecular electrostatic potential (MEP) of **TS3** (blue) and in **TS2** (green) as a consequence of the larger positive charge developed in the former).

## 5. Molecular Electron Density Theory (MEDT)

Numerous works performed in the last decade devoted to the study of the molecular reactivity in relevant organic reactions based on the quantum topological analysis of the changes in electron density along a reaction path have allowed proposing a new theory for the study of the molecular reactivity in organic reactions, named Molecular Electron Density Theory (MEDT). This theory is based on the idea that while the electron density distribution at the GS is responsible for physical and chemical molecular properties, as proposed by DFT [19], the capability for changes in electron density is responsible for molecular reactivity [61]. Accordingly, molecular reactivity in Organic Chemistry cannot be characterised only by a simple energy and geometrical study of the corresponding stationary points, but by a quantum chemical analysis of the changes of electron density as well as the energies associated with these changes along the reaction path, in order to understand experimental outcomes and explain the molecular mechanism. Thus, MEDT is presented as an insightful new theory in the study of organic reactions.

Besides the exploration and characterisation of the potential energy surface (PES) associated with the studied reaction, within MEDT, the molecular reactivity in organic reactions is studied using quantum chemical tools based on the analysis of the electron density such as the analysis of the conceptual DFT reactivity indices at the GS of the molecules [76,77], the ELF topological analysis [35,36,37,38,39] of the electron density focused on the progress of the bonding changes along the reaction path, and the non-covalent interactions [85] (NCI) analysis of the electron density at the TSs in order to characterise weak interactions determining the selectivity in organic reactions.

**Figure 6:**
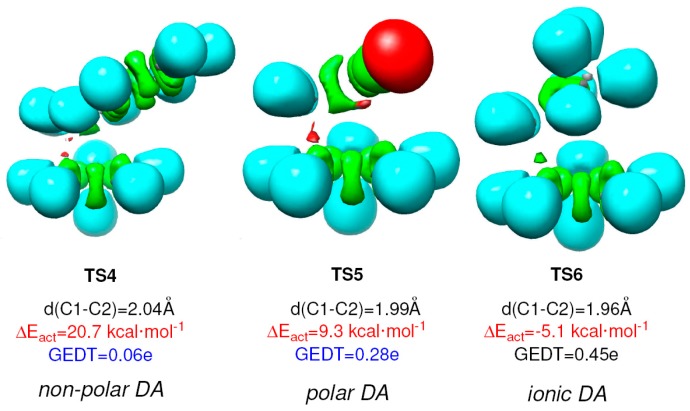
B3LYP/6-31G(d) ELF valence basins, C–C distances, activation energies and GEDT of the TSs involved in the non-polar, polar and ionic DA reactions of Cp **6** with styrene **10**, **TS4**, dicyanoethylene **11**, **TS5**, and iminium cation **12**, **TS6**.

This methodology, which has been applied in recent theoretical studies [61,86,87,88], allows introducing new chemical concepts and reactivity models that make it possible to establish a modern rationalisation and to gain insight into molecular mechanisms and reactivity in Organic Chemistry. Within this MEDT it is important to highlight the useful classification of DA reactions into non-polar, polar and ionic reactions [65]. As can be seen in Figure 6, and as has been commented in Section 4, the increase of the polar character of the DA reaction, i.e., the GEDT, decreases the corresponding activation energy, the nucleophilic/electrophilic interactions at the TSs being the main factor controlling the DA reactions [65]. Figure 6 also shows that while at **TS4** and **TS5** the C–C single bond is not yet formed, at the more slightly advanced **TS6**, the formation of the new C–C single bond has already begun [61].

**Figure 7:**
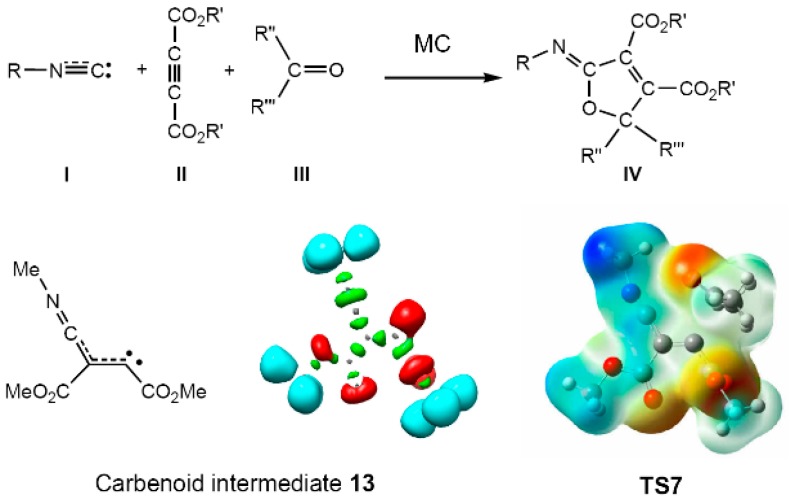
ELF valence basins of the highly nucleophilic carbenoid intermediate **13** and the MEP of **TS7**, associated with the nucleophilic attack of carbenoid intermediate **13** on ketone. ELF valence basins were represented at an isosurface value of 0.8. Calculations were performed at the MPWB1K/6-311G(d,p) level.

The study of the high reactivity of the carbenoid intermediate **13** [86] also deserves to be mentioned as an important contribution of MEDT to organic chemical reactivity (see Figure 7). It was proposed that zwitterionic intermediates generated in the multicomponent (MC) reactions between isocyanides **I** and dialkyl acetylenedicarboxylates **II** react quickly with carbonyl compounds **III** in a 1,3-dipolar cycloaddition. A recent MEDT study for the high reactivity of these intermediates has shown that their strong carbenoid character together with the special molecular structure of this intermediate that favours the approach of the electrophilic carbonyl compound to these carbenoid intermediates is responsible for their high nucleophilic reactivity [86] (see the MEP of **TS7**, associated with the nucleophilic attack of carbenoid intermediate **13** on ketone in Figure 7).

**Scheme 3:**
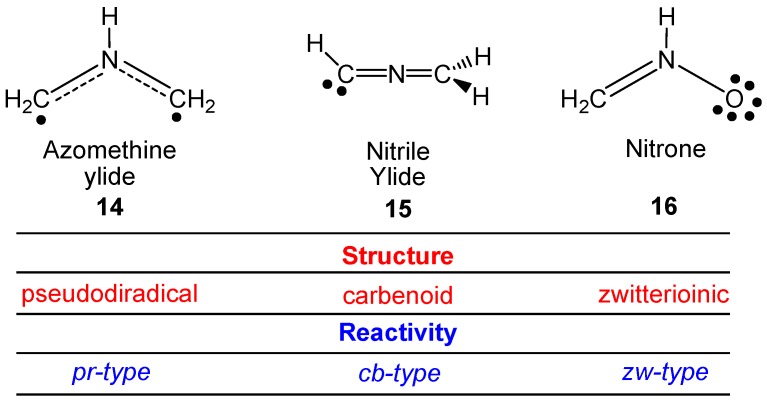
Electronic structure of TACs and the proposed reactivity types in 32CA reactions.

Finally, the applicability of MEDT in organic reactivity is also emphasized through the useful classification of the mechanisms of 32CA reactions into pseudodiradical-type [87] (*pr-type*), carbenoid-type [88] (*cb-type*) and zwitterionic-type [87] (*zw-type*) 32CA reactions (see Scheme 3). 32CA reactions are among the most powerful methods for the synthesis of five-membered heterocyclic compounds since 1961, when Huisgen developed his mechanistic study [89]. TACs with a pseudodiradical character, such as azomethyne ylide **14**, participate in *pr-type* 32CA reactions taking place easily through earlier TSs with a very low polar character [87,90] and TACs with a carbenoid character, such as nitrile ylide **15**, or zwitterionic character, such as nitrone **16**, participate in *cb-type* [88,91] or *zw-type* [87,92] 32CA reactions, respectively, whose feasibility depends on the polar character of the reaction, i.e., the nucleophilic character of the TAC and the electrophilic character of the ethylene derivative.

Note that in Huisgen’s seminal study, all TACs, named 1,3-dipoles, were represented by 1,2-zwitterionic structures and the reactions were named 1,3-dipolar cycloadditions [80,93]. However, based on ELF topological analysis and NPA of some TACs, it appears that their structure and reactivity are neither related to 1,3- nor 1,2-zwitterionic species, especially pseudodiradical [81] and carbenoid [91] TACs.

## 6. Conclusions

Since the establishment of chemistry as a branch of scientific knowledge, Physical and Organic Chemistry have grown separately. Although a large community of physical chemists has widely made use of the analysis of the electron density in the study of chemical reactivity since the development of computational resources, Organic Chemistry has remained at a standstill with concepts derived from the FMO theory. MEDT is not an assertion of the scope of the already known electron density based methodologies within Organic Chemistry, but a theoretical alternative to study reactivity in Organic Chemistry.

MEDT is based on the idea that while the electron density distribution at the GS is responsible for physical and chemical molecular properties, as proposed in DFT [19], the capability for changes in electron density, and not MO interactions, is responsible for molecular reactivity [61]. Reactivity in Organic Chemistry cannot be characterised by simple energy and geometrical studies of the corresponding stationary points, but by a quantum chemical analysis of the changes of electron density as well as the energies associated with these changes along the reaction path, in order to understand experimental outcomes.

The proposed molecular reactivity theory based on changes of the electron density, and not on MO interactions, allows introducing new chemical concepts and reactivity models, making it possible to establish a modern rationalisation and to gain insight into molecular mechanisms and reactivity in Organic Chemistry.

## 7. Computational Methods

All calculations were performed using *ab initio* HF theory [18] or DFT using the B3LYP [24,25], the MPWB1K [26] or the M06-2X [27] functionals together with the standard 6-31G(d) basis set [18]. TSs were characterised by frequency computations in order to verify that they have one and only one imaginary frequency. The GEDT [61] at the TSs is computed by sharing the natural atomic charges, obtained by NPA [54] at the TSs, between the nucleophilic and the electrophilic frameworks. GEDT values are hardly dependent on the computational method used to obtain the atomic charges, as it comes mainly from the integration of the electron density of two molecular frameworks that are usually not yet bound at the TSs [67]. The topological analysis of the ELF, *η*(**r**) [35], was performed with the TopMod program [94] using the corresponding *ab initio* or DFT mono-determinantal wavefunctions. All computations were carried out with the Gaussian 09 suite of programs [95].

## Figures and Tables

**Table 1:** Activation and reaction energies ^a^, in kcal·mol^−1^, and C–C distances of the two forming single bonds, in Angstroms, at **TS1**, obtained using different computational levels.

Method	ΔE_act_	ΔE_reac_	d(C–C)
Experimental	27.5	−38.4	
HF/6-31G(d)	47.4	−36.0	2.201
MP2/6-31G(d)	20.0	−45.9	2.286
B3LYP/6-31G(d)	24.8	−32.9	2.273
MPWB1K/6-31G(d)	22.1	−57.7	2.252
M06-2X/6-31G(d)	19.4	−43.5	2.269

^a^ Relative energies include zero point energies.
